# A critical bioenergetic switch is regulated by IGF2 during murine cartilage development

**DOI:** 10.1038/s42003-022-04156-4

**Published:** 2022-11-11

**Authors:** Judith M. Hollander, Lingyun Li, Miraj Rawal, Si Kun Wang, Yue Shu, Ming Zhang, Heber C. Nielsen, Clifford J. Rosen, Li Zeng

**Affiliations:** 1 Program in Cell, Molecular and Developmental Biology, Graduate School of Biomedical Sciences, Tufts University, 136 Harrison Avenue, Boston, MA 02111 USA; 2 Department of Immunology, Tufts University School of Medicine, 136 Harrison Avenue, Boston, MA 02111 USA; 3 Program in Pharmacology, Graduate School of Biomedical Sciences, Tufts University, 136 Harrison Avenue, Boston, MA 02111 USA; 4 Department of Computer Science, Boston University, Boston, MA 02215 USA; 5 Department of Pediatrics, Tufts Medical Center, 800 Washington Street, Boston, MA 02111 USA; 6 Center for Molecular Medicine, Maine Medical Center Research Institute, Scarborough, ME 04074 USA; 7 Department of Orthopaedics, Tufts Medical Center, 800 Washington Street, Boston, MA 02111 USA

**Keywords:** Cartilage development, Cartilage

## Abstract

Long bone growth requires the precise control of chondrocyte maturation from proliferation to hypertrophy during endochondral ossification, but the bioenergetic program that ensures normal cartilage development is still largely elusive. We show that chondrocytes have unique glucose metabolism signatures in these stages, and they undergo bioenergetic reprogramming from glycolysis to oxidative phosphorylation during maturation, accompanied by an upregulation of the pentose phosphate pathway. Inhibition of either oxidative phosphorylation or the pentose phosphate pathway in murine chondrocytes and bone organ cultures impaired hypertrophic differentiation, suggesting that the appropriate balance of these pathways is required for cartilage development. Insulin-like growth factor 2 (IGF2) deficiency resulted in a profound increase in oxidative phosphorylation in hypertrophic chondrocytes, suggesting that IGF2 is required to prevent overactive glucose metabolism and maintain a proper balance of metabolic pathways. Our results thus provide critical evidence of preference for a bioenergetic pathway in different stages of chondrocytes and highlight its importance as a fundamental mechanism in skeletal development.

## Abstract (web summary)

Murine chondrocyte hypertrophy was found to require IGF2-regulated bioenergetic reprogramming, in which glucose utilization shifts from glycolysis to oxidative phosphorylation as chondrocytes mature.

## Introduction

During development, long bones form through endochondral ossification. The central feature of this process is the development of a cartilage template, known as the growth plate, which drives longitudinal growth through chondrocyte proliferation, hypertrophy, and ultimately differentiation into osteoblasts^1–8^. The timing of the transition from proliferation to hypertrophy is controlled by the parathyroid hormone-related protein (PTHrP)/Indian hedgehog (Ihh) signaling loop, as well as other signals such as growth hormone (GH), insulin-like growth factor 1 (IGF1), bone morphogenetic proteins (BMPs), ascorbic acid, and reactive oxygen species (ROS)^1,9–14^. Disruption of the transition from proliferation to hypertrophy leads to growth abnormalities, such as dwarfism and gigantism, indicating that the timing for this transition is critical to bone development.

Silver-Russell Syndrome (SRS) is a genetic condition characterized by postnatal growth restriction and craniofacial abnormalities. It occurs in 1 in 3000 to 1 in 100,000 live births^15–17^. It was not until recently that nonsense *IGF2* mutations were discovered in these patients, revealing a role for IGF2 in postnatal skeletal growth^18,19^. Interestingly, children with IGF2 mutations had near normal levels of IGF1^18^. Furthermore, growth hormone treatment was only effective in some SRS patients, suggesting a GH/IGF1-independent mechanism of growth restriction^18^. To elucidate how IGF2 regulates postnatal skeletal growth, we characterized the *Igf2* null mouse, which recapitulates the postnatal growth restriction characteristic of SRS. In the absence of IGF2, chondrocytes exhibited aberrant hypertrophy, indicating that IGF2 is critical for hypertrophic differentiation. Unexpectedly, we found that *Igf2* null chondrocytes had a higher overall glucose metabolic activity than WT chondrocytes^20^. However, although it has been established that glucose is utilized in growth plate chondrocytes^21^, the significance of glucose metabolic regulation in normal cartilage development is still largely elusive, making it difficult to fully comprehend how IGF2 may regulate this process.

Typically, glucose is metabolized via glycolysis or oxidative phosphorylation (OxPhos). Alternatively, it is shunted into the pentose phosphate pathway (PPP) for production of nucleic acid backbones and the generation of NADPH, which is vital for protein and lipid synthesis and redox equilibrium. The balance of these pathways constitutes a metabolic signature whose maintenance is critical for the identity and function of each cellular stage in tissues throughout the body^22–30^. In this study, we discovered that normal chondrocyte hypertrophy is marked by a bioenergetic shift in the equilibrium of glycolysis and oxidative phosphorylation, which is further balanced by the PPP. This equilibrium is critical for proper chondrocyte maturation and is regulated by IGF2 in a stage-specific manner.

## Results

### Hypertrophy is marked by a profound increase in oxidative phosphorylation that is regulated by IGF2

**Fig. 1:**
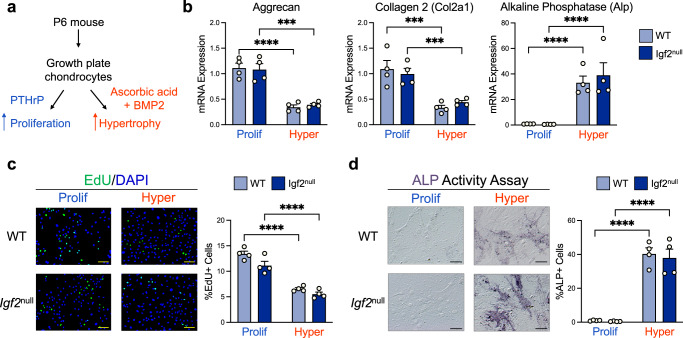
Murine epiphyseal chondrocytes can be enriched to proliferating and hypertrophic phenotypes. **a** Schematic of the experimental design used to enrich populations of chondrocytes to proliferating (“Prolif”) and hypertrophic (“Hyper”) phenotypes. **b** Gene expression of aggrecan, collagen 2 (proliferation marker genes), and alkaline phosphatase (hypertrophic marker gene) was measured by RT-qPCR in proliferating and hypertrophic epiphyseal chondrocytes from WT and *Igf2* null mice. **c** Assessment of proliferating chondrocytes by EdU staining. **d** Assessment of chondrocyte hypertrophy by alkaline phosphatase activity staining. Scale bar: 100 µm. Data are presented as individual measurements with mean + SEM, representative of *n* = 5 experiments with 2–4 wells per group. Statistical significance was calculated using two-way ANOVA with Tukey’s test for multiple comparisons (**b**, **c**, **d**). *p*-values indicated as *p < 0.05, **p < 0.01, ***p < 0.001, ****p < 0.0001.

To determine whether chondrocytes have distinct metabolic signatures during cartilage development, we first enriched postnatal growth plate chondrocytes to proliferating and hypertrophic stages for subsequent bioenergetic investigations. Based on prior knowledge, we treated chondrocytes with PTHrP to maintain a proliferating phenotype, or with a combination of ascorbic acid (AA) and bone morphogenetic protein 2 (BMP2) to induce hypertrophy (Fig. 1a)^11,31,32^. Since we previously determined that *Igf2* null chondrocytes have overactive glucose metabolism, we included both WT and *Igf2* null chondrocytes in this investigation^20^. As expected, chondrocytes treated with PTHrP expressed high levels of the proliferative marker genes aggrecan and collagen 2, and thus are labeled as “proliferating” in this article. Chondrocytes treated with AA + BMP2, on the other hand, showed decreased aggrecan and collagen 2 mRNA expression and increased expression of the hypertrophic marker alkaline phosphatase (*Alp*), and are referred to as “hypertrophic” (Fig. 1b). Maintenance of proliferation by PTHrP and induction of hypertrophy by AA + BMP2 were further verified by EdU staining and ALP activity assays (Fig. 1c, d). No significant differences in the expression of proliferative or hypertrophic markers were found between WT and *Igf2* null chondrocytes, suggesting we were able to enrich all chondrocytes to the same degree. Therefore, the metabolic profiles obtained from our analyses are unlikely to be due to skewed representation of proliferating or hypertrophic cells in isolated WT and *Igf2* null chondrocytes.

**Fig. 2:**
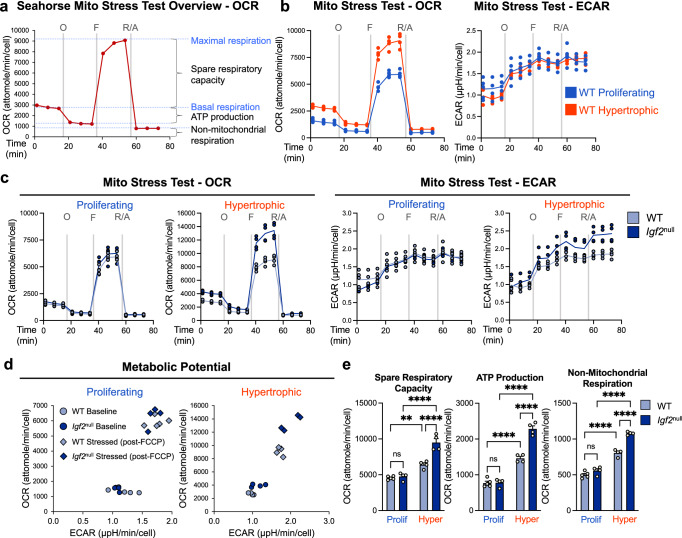
Hypertrophic differentiation is characterized by a marked increase in oxidative phosphorylation that is further exacerbated by *Igf2* deficiency. **a** Schematic of expected oxygen consumption rate (OCR) readings for a Mito Stress Test in the Seahorse XFe96 metabolic analyzer, including injection points for oligomycin (O), FCCP (F), and rotenone/antimycin A (R/A). **b** Metabolic analysis: OCR and extracellular acidification rate (ECAR) of WT epiphyseal chondrocytes under proliferative and hypertrophic conditions. Note that the OCR curve for WT hypertrophic chondrocytes also appears in (**a**). **c** OCR and ECAR analyses of WT and *Igf2* null epiphyseal chondrocytes under proliferative and hypertrophic conditions. Note that the WT lines in (**c**) are the same as those in (**a**) and (**b**). **d** Metabolic potential of WT and *Igf2* null epiphyseal chondrocytes at baseline and after being stressed (post-FCCP injection). **e** Spare respiratory capacity, (mitochondrial) ATP production, and non-mitochondrial respiration as calculated from OCR measurements. Data are presented as individual measurements with a line indicating the mean where necessary (**b**, **c**, **d**) or individual measurements with mean + SEM (**e**), representative of *n* = 5 experiments with 3–6 wells per group. Statistical significance was calculated using a two-way ANOVA with Tukey’s test for multiple comparisons (**e**). *p*-values indicated as ns (not significant), *p < 0.05, **p < 0.01, ***p < 0.001, ****p < 0.0001.

To understand how glucose is metabolized during chondrocyte differentiation, we used the Seahorse metabolic analyzer. We measured glycolysis and oxidative phosphorylation (OxPhos) in the above enriched proliferating and hypertrophic populations, capitalizing on the Seahorse’s ability to measure these processes in live cells and in real time. Since OxPhos takes place in the mitochondria, we performed the Seahorse Mito Stress Test, which primarily aims to evaluate OxPhos activity through measurement of the Oxygen Consumption Rate (OCR). In this test, addition of oligomycin, an inhibitor of ATPase (Complex V) in the electron transport chain (ETC), is expected to reduce OCR, allowing for the calculation of mitochondrial ATP production. FCCP, an uncoupler of the ETC, is expected to maximize mitochondrial OCR, from which the spare respiratory capacity is deduced. Finally, rotenone and antimycin A (R/A), inhibitors of complexes I and III of the ETC, respectively, completely block mitochondrial respiration, and thus any component of the OCR derived from the mitochondria. What remains after R/A treatment indicates the non-mitochondrial respiration (Fig. 2a).

We first evaluated OCR profiles in WT chondrocytes to characterize OxPhos during normal hypertrophy. Compared with WT proliferating chondrocytes, WT hypertrophic chondrocytes exhibited a higher OCR at baseline, as well as higher OCR after each inhibitor treatment (Fig. 2b). In the meantime, Extracellular Acidification Rate (ECAR) exhibited an opposite trend, in which hypertrophic chondrocytes seem to have a lower baseline level (Fig. 2b). We next compared WT chondrocytes to *Igf2* null chondrocytes at each stage of differentiation. Strikingly, *Igf2* null chondrocytes had an even higher OCR than WT under hypertrophic conditions, though no difference was seen between them under proliferative conditions (Fig. 2c). We then plotted the metabolic potential of WT and *Igf2* null chondrocytes based on the OCR and ECAR at baseline or after addition of FCCP, which represents a stressed (uncoupled) condition (Fig. 2d). The resulting plot further illustrates the higher aerobic and glycolytic potential in the *Igf2* null hypertrophic chondrocytes when stressed^33,34^. Accordingly, hypertrophic chondrocytes had a significantly higher spare respiratory capacity, ATP production, and non-mitochondrial respiration than their proliferating counterparts (Fig. 2e). The differences in these metrics were even more pronounced in the *Igf2* null hypertrophic chondrocytes. Our results therefore suggest that hypertrophic chondrocytes have a higher level of OxPhos activity than proliferating chondrocytes. The lack of IGF2 caused a further increase in mitochondrial ATP production and spare respiratory capacity upon hypertrophy, suggesting that IGF2 prevents overactive OxPhos activity in hypertrophic chondrocytes.

### Increased oxidative phosphorylation during hypertrophy is accompanied by decreased glycolysis

**Fig. 3:**
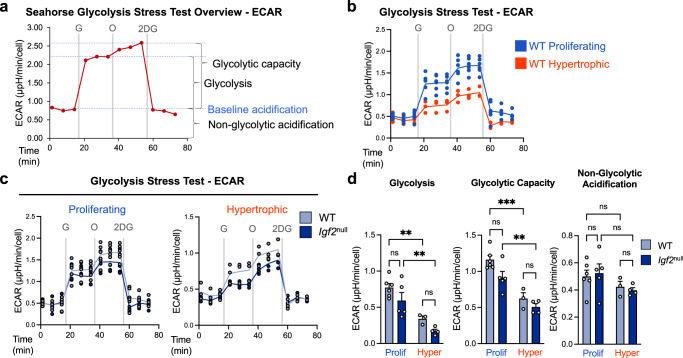
Hypertrophy is characterized by a decrease in glycolysis and glycolytic capacity. **a** Schematic of expected extracellular acidification rate (ECAR) readings for a Glycolysis Stress Test in the Seahorse XFe96 metabolic analyzer, including injection points for glucose (G), oligomycin (O), and 2-deoxyglucose (2DG). **b** Metabolic analysis (ECAR) of WT epiphyseal chondrocytes under proliferative and hypertrophic conditions. **c** Metabolic analysis of WT and *Igf2* null epiphyseal chondrocytes under proliferative and hypertrophic conditions. Note that the WT lines in (**c**) are the same as in (**b**). **d** Glycolysis, glycolytic capacity, and non-glycolytic acidification as calculated from ECAR measurements. Data are presented as individual measurements with a line indicating the mean (**b**, **c**) or individual measurements with mean + SEM (**d**), representative of *n* = 4 experiments with 3–6 wells per group. Statistical significance was calculated using a two-way ANOVA with Tukey’s test for multiple comparisons (**d**). *p*-values indicated as ns (not significant), *p < 0.05, **p < 0.01, ***p < 0.001.

In addition to acidity from lactate production during glycolysis, acidity from CO_2_ made during respiration can also contribute to ECAR (Fig. 2b–d). Therefore, to directly evaluate glycolysis and glycolytic capacity in these cells, we employed the more suitable Glycolysis Stress Test assay (Fig. 3a). In this assay, ECAR from glucose-starved chondrocytes (baseline) is expected to increase upon administration of glucose, and further increase when OxPhos is inhibited by oligomycin. Reduction of ECAR to baseline after addition of 2-deoxyglucose (2DG, an analog of glucose that cannot be metabolized) confirms that the baseline ECAR was due to non-glycolytic acidification (Fig. 3a).

We observed no significant difference in baseline ECAR between proliferating and hypertrophic states in WT cells (Fig. 3b). However, administration of glucose and oligomycin both led to a large increase in ECAR in proliferating cells, but only a moderate increase in hypertrophic cells (Fig. 3b). This trend was the same in *Igf2* null chondrocytes (Fig. 3c). Therefore, proliferating chondrocytes use glycolysis more readily than hypertrophic chondrocytes, consistent with the previous analysis showing a higher level of OxPhos in hypertrophic chondrocytes (Fig. 2c). Compared to WT, *Igf2* null chondrocytes tended to have lower ECAR than WT in response to both glucose and oligomycin (Fig. 3c). Further analysis of the Glycolysis Stress Test data confirmed a significant decrease in both glycolysis and glycolytic capacity in hypertrophic chondrocytes regardless of the genotype (Fig. 3d).

Taken together, we conclude that chondrocytes at different developmental stages have distinct metabolic signatures. Compared to proliferating chondrocytes, hypertrophic chondrocytes have a lower level of glucose utilization through glycolysis, and a higher level of utilization through OxPhos, suggesting that there is a bioenergetic switch from glycolysis to OxPhos during normal development.

### Oxidative phosphorylation is required for normal hypertrophic differentiation

We next investigated whether the substantial increase in OxPhos in the hypertrophic stage has any functional significance in terms of cartilage development, or merely represents a casual association. To distinguish between the two possibilities, we inhibited OxPhos to assess its requirement in cartilage development.

**Fig. 4:**
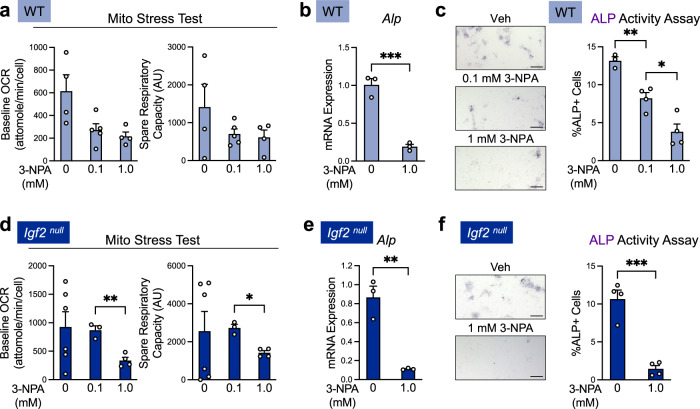
Inhibition of oxidative phosphorylation in normal chondrocytes strongly inhibits hypertrophy in cultured epiphyseal chondrocytes. **a** Baseline and spare respiratory OCR measurements from Mito Stress Tests of WT hypertrophic epiphyseal chondrocytes treated with SDH inhibitor 3-NPA, showing that 3-NPA inhibits OxPhos. Representative of *n* = 3 experiments with 3–6 wells per group. **b** RT-qPCR analysis of hypertrophic marker alkaline phosphatase (ALP) in WT hypertrophic epiphyseal chondrocytes treated with 3-NPA. Representative of *n* = 2 experiments with 3 wells per group. **c** Alkaline phosphatase activity assay of WT hypertrophic chondrocytes treated with 3-NPA. Representative of *n* = 3 experiments with 2–4 wells per group. Scale bar: 200 µm. **d** Baseline and spare respiratory capacity OCR measurements of *Igf2* null hypertrophic epiphyseal chondrocytes treated with 3-NPA. Representative of *n* = 3 experiments with 3–6 wells per group. **e** Gene expression of hypertrophic marker alkaline phosphatase as measured by RT-qPCR in *Igf2* null hypertrophic epiphyseal chondrocytes treated with 3-NPA. Representative of *n* = 2 experiments with 3 wells per group. **f** *Igf2* null hypertrophic chondrocytes treated with 3-NPA were detected and quantified by staining for alkaline phosphatase activity. Representative of *n* = 3 experiments with 2–4 wells per group. Scale bar: 200 µm. Data are presented as individual measurements with mean + SEM. Note that the control data (0 mM 3-NPA) for **b** and **e** are also shown in Fig. 6. Statistical significance was calculated by Brown–Forsythe and Welch ANOVAs with Dunnett’s T3 test for multiple comparisons (**a**, **c**, **d**) or unpaired two-tailed *t*-test (**b**, **e**, **f**). *p*-values indicated as *p < 0.05, **p < 0.01, ***p < 0.001.

We chose 3-nitropropionic acid (3-NPA), a widely used and highly specific inhibitor of succinate dehydrogenase (SDH), to accomplish this. As SDH is a key enzyme in both the tricarboxylic acid (TCA) cycle and complex II in the ETC, 3-NPA treatment efficiently inhibits OxPhos^35,36^. Because completely blocking respiration has the potential to cause substantial cell death if sufficient amounts of the inhibitor are used, we first performed live-dead assays on chondrocytes and TUNEL assays on cultured metatarsal bones, using the 3-NPA concentrations commonly reported in other investigations^35,36^. Our results indicate that if 3-NPA is used at concentrations less than 1.0 mM in chondrocyte cultures, the level of cell death is minimal (Supplementary Fig. 1a). We then used a Mito Stress Test to confirm that 3-NPA, when used at 1.0 mM or lower, could still inhibit OxPhos. Indeed, we observed OCR levels trending down upon 3-NPA treatment (Fig. 4a). This dose selection process allowed us to evaluate the effect of OxPhos reduction in a dose-dependent way without causing extensive cell death.

Because we observed a marked increase in OxPhos in enriched hypertrophic chondrocytes (Fig. 2b, c), we first evaluated the effect of 3-NPA on WT cells. Strikingly, 3-NPA led to a substantial decrease in the mRNA expression and enzyme activity of hypertrophic marker alkaline phosphatase (ALP), as compared to vehicle (DMSO) treatment (Fig. 4b, c). Since we observed that OxPhos was strongly upregulated in *Igf2* null cells, we next evaluated whether blocking it would have the same effect in *Igf2* null chondrocytes as it did in WT. Indeed, 3-NPA reduced baseline OCR and spare respiratory capacity in the Mito Stress Test, but only at the concentration of 1.0 mM (Fig. 4d). Thus, we used 1.0 mM 3-NPA to evaluate the effect on chondrocyte hypertrophy and found that it decreased ALP mRNA and activity levels in *Igf2* null cells, as it did in WT cells (Fig. 4e, f).

**Fig. 5:**
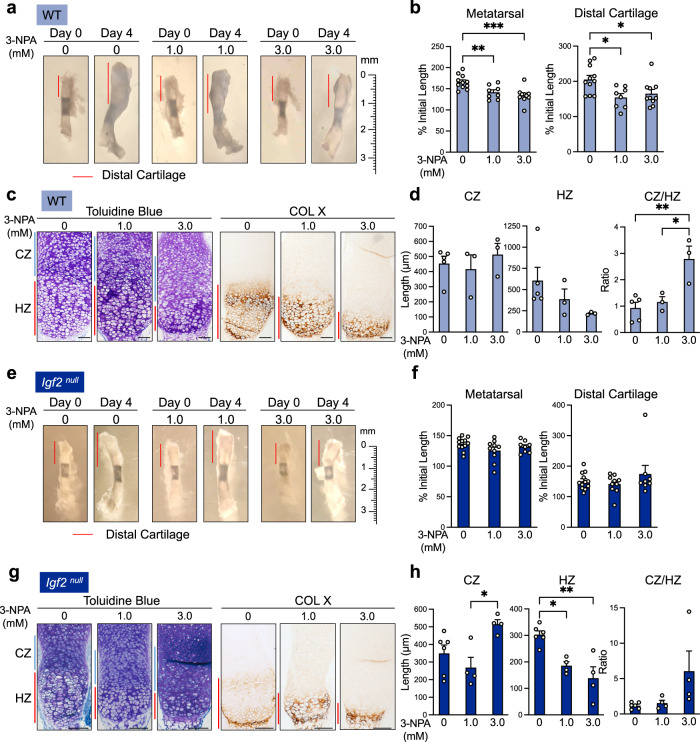
Inhibition of oxidative phosphorylation in ex vivo metatarsal culture does not alter bone growth, but increases the length of the columnar zone at the expense of the hypertrophic zone. **a** Images of WT metatarsal explants cultured for 4 days with and without 3-NPA. Representative of *n* = 8–11 bones per group. **b** Quantification of metatarsal and distal cartilage growth from the beginning of the culture period (as percent of initial length of metatarsal or distal cartilage). *n* = 8–11 bones per group. **c** Toluidine blue staining and collagen X (hypertrophic marker) IHC on ex vivo-cultured WT metatarsals treated with 3-NPA. Representative of *n* = 3–5 bones per group. CZ columnar zone. HZ hypertrophic zone. **d** Measurements of the lengths of the CZ and HZ, and the ratio of the CZ to the HZ, in ex vivo-cultured WT metatarsals treated with 3-NPA. *n* = 3–5 bones per group. **e** *Igf2* null metatarsal explants cultured for 4 days with and without 3-NPA. Representative of *n* = 8–12 bones per group. **f** Quantification of metatarsal and distal cartilage growth from the beginning of the culture period (as percent of initial length of metatarsal or distal cartilage). *n* = 8–12 bones per group. **g** Toluidine blue staining for morphology and IHC for collagen X (hypertrophic marker) in *Igf2* null metatarsals treated with 3-NPA. Representative of *n* = 4–6 bones per group. CZ columnar zone. HZ hypertrophic zone. **h** Measurements of the lengths of the CZ and HZ, and the ratio of the CZ to the HZ, in *Igf2* null metatarsals treated with 3-NPA. *n* = 4–6 bones per group. Scale bar: 100 µm. Data are presented as individual measurements with mean + SEM. Note that the control data (0 mM 3-NPA) for **b**, **d**, **f**, and **h** are also shown in Fig. 7. Statistical significance was calculated one-way ANOVA with Sidak’s test for multiple comparisons (**b**, **d**, **f**, **h**). *p*-values indicated as *p < 0.05, **p < 0.01, ***p < 0.001.

We next applied 3-NPA to cultured metatarsal bones, which we and others have used as an ex vivo model of cartilage development and long bone growth^20,37–41^. Considering metatarsal bone cultures might require a higher concentration of 3-NPA than the two-dimensional chondrocyte cultures in our in vitro study, we used 1.0 and 3.0 mM 3-NPA for adequate tissue penetration. Under vehicle-treated (DMSO) conditions, these bones increased in length by nearly 70% in 4 days, and the length of the distal cartilage was doubled (Fig. 5a). Treatment with 3-NPA caused an almost 50% reduction in metatarsal bone and distal cartilage growth (Fig. 5a, b). Toluidine blue staining indicated that inhibition of OxPhos altered the length of the hypertrophic zone (HZ) with respect to the columnar zone (CZ) (Fig. 5c, d). To further define the HZ, we performed collagen X (Col X) immunohistochemistry (IHC) and confirmed that there was a drastic reduction in the length of the HZ in metatarsals treated with 3-NPA. Once quantified, we found no significant change in the length of the CZ, but a trend of substantial decrease in the HZ. As a result, there was a dose-dependent increase in the ratio of CZ/HZ by 3-NPA (Fig. 5d). We confirmed that these doses of 3-NPA did not cause cell death using TUNEL staining (Supplementary Fig. 1b). This result thus suggests that OxPhos is required for the progression of chondrocytes from the columnar proliferating stage to the hypertrophic stage. We also used another set of OxPhos inhibitors, rotenone and antimycin A (R/A). R/A is well known to inhibit complexes I and III in the ETC and thus OCR (Fig. 2). We observed that R/A similarly decreased ALP activity and hypertrophic zone length as 3-NPA did (Supplementary Fig. 2), confirming the specificity of our results.

We then investigated the effect of OxPhos inhibition in ex vivo cultures of *Igf2* null metatarsals (Fig. 5e). Over the course of 4 days, *Igf2* null bones grew by just 35% and distal cartilage by 50% (Fig. 5e, f). This is much slower than the WT growth rate, which is consistent with the results of our previous comparison of the in vivo growth of WT and *Igf2* null bones^20^. Toluidine blue staining and Col X IHC revealed an expanded CZ and a reduced HZ in bones treated with 3.0 mM 3-NPA, increasing the ratio of CZ/HZ and suggesting a halt in the shift from the columnar stage to the hypertrophic stage (Fig. 5g, h). Additionally, the expanded length of the CZ in *Igf2* null bones treated with 3.0 mM 3-NPA was similar to the length of the CZ in vehicle-treated WT bones, further suggesting that the shift from the columnar stage to the hypertrophic stage is halted during OxPhos inhibition. Taken together, these data suggest that OxPhos, regulated by IGF2, is required for proper chondrocyte transition to hypertrophic differentiation.

### The pentose phosphate pathway is upregulated during chondrocyte maturation and is required for normal hypertrophic differentiation

In addition to glycolysis and OxPhos, the PPP is another major pathway in glucose metabolism, and is reported to be induced by increased ROS to combat oxidative stress^42,43^. Because our results suggest hypertrophy is accompanied by an increase in OxPhos and ROS are known to be elevated in the hypertrophic zone, we next investigated whether the PPP is also differentially regulated during chondrocyte maturation. Prior reports in rat and chick models have shown increased activity of glucose-6-phosphate dehydrogenase (G6PD) in hypertrophic cartilage^44,45^. This enzyme catalyzes the rate-limiting step of the PPP, and thus has been widely used as a readout of this pathway^46,47^. However, a role for the PPP during cartilage development has not been reported.

**Fig. 6:**
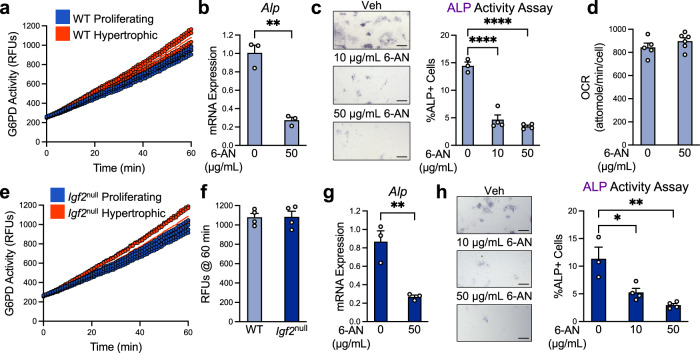
The pentose phosphate pathway is required for normal hypertrophy in cultured epiphyseal chondrocytes. **a** G6PD activity measurements in WT epiphyseal chondrocytes under proliferative and hypertrophic conditions. Representative of *n* = 3 experiments with 3–4 wells per group. **b** Gene expression of hypertrophic marker alkaline phosphatase (*Alp*) as measured by RT-qPCR in WT hypertrophic epiphyseal chondrocytes treated with G6PD inhibitor 6-AN. Representative of *n* = 2 experiments with 3 wells per group. **c** WT hypertrophic chondrocytes treated with 6-AN were assessed by staining for alkaline phosphatase (ALP) activity. Representative of *n* = 3 experiments with 2–4 wells per group. Scale bar: 200 µm. **d** Baseline OCR measurements of WT epiphyseal chondrocytes under proliferative and hypertrophic conditions and treated with PPP inhibitor 6-AN. Representative of *n* = 3 experiments with 3–6 wells per group. **e** G6PD activity in *Igf2* null epiphyseal chondrocytes under proliferative and hypertrophic conditions. Representative of *n* = 3 experiments with 3-4 wells per group. **f** G6PD activity measurements in WT and *Igf2* null epiphyseal chondrocytes under hypertrophic conditions. **g** Gene expression of hypertrophic marker alkaline phosphatase *Alp* as measured by RT-qPCR in *Igf2* null hypertrophic epiphyseal chondrocytes treated with 6-AN. Representative of *n* = 2 experiments with 3 wells per group. **h** *Igf2* null hypertrophic chondrocytes treated with 6-AN were assessed by staining for ALP activity. Representative of *n* = 3 experiments with 2–4 wells per group. Scale bar: 200 µm. Data are presented as individual data points with a line indicating the mean (**a**, **e**) or as individual measurements with mean + SEM (**b**, **c**, **d**, **f**, **g**, **h**). Note that the control data (0 µg/mL 6-AN) for **b** and **g** are also shown in Fig. 4. Statistical significance was calculated by unpaired two-tailed *t*-test (**b**, **d**, **f**, **g**) or one-way ANOVA with Tukey’s test for multiple comparisons (**c**, **h**). *p*-values indicated as *p < 0.05, **p < 0.01, ***p < 0.001, ****p < 0.0001.

We first assayed G6PD in enriched murine growth plate chondrocyte populations and confirmed that hypertrophic chondrocytes also had elevated G6PD activity compared to proliferating chondrocytes (Fig. 6a). We then determined whether reducing PPP activity would affect hypertrophic differentiation. We selected the widely used inhibitor 6-aminonicotinamide (6-AN), which acts as a competitive inhibitor of the PPP by blocking generation of NADPH from NADP^+^^48^. We first established that if a dose of 6-AN below 50 µg/mL was used, the toxicity was minimal in cultured chondrocytes (Supplementary Fig. 3a). In growth plate chondrocytes undergoing hypertrophy, we found that 50 µg/mL of 6-AN significantly reduced the level of ALP mRNA and enzyme activity, suggesting that it inhibited hypertrophy (Fig. 6b, c). Because this result was very similar to that of OxPhos inhibition (Fig. 4), we evaluated OCR in these cells. We found that 6-AN did not cause any changes in baseline OCR, thus excluding the possibility that the reduction in hypertrophic differentiation observed here was due to a reduction in OxPhos (Fig. 6d).

We further investigated whether the PPP could be similarly upregulated in *Igf2* null chondrocytes to balance the increased OxPhos in these mutant chondrocytes. We observed that G6PD activity was increased in the hypertrophic population of *Igf2* null cells as it was in the hypertrophic population of WT cells (Fig. 6e). However, when compared to WT hypertrophic chondrocytes, *Igf2* null hypertrophic chondrocytes had the same level of G6PD activity (Fig. 6f). Exogenous IGF2 treatment only slightly lowered G6PD activity, suggesting that IGF2 does not play a critical role in the regulation of the PPP (Supplementary Fig. 4). Treatment of *Igf2* null hypertrophic chondrocytes with 6-AN caused a significant decrease in both the mRNA expression and activity level of ALP, suggesting that inhibition of the PPP led to suppressed hypertrophy in these cells (Fig. 6g, h). Thus, although the level of the PPP was not correspondingly increased to match the increase in OxPhos in *Igf2* null cells, the PPP is still required for chondrocyte hypertrophy in these cells.

**Fig. 7:**
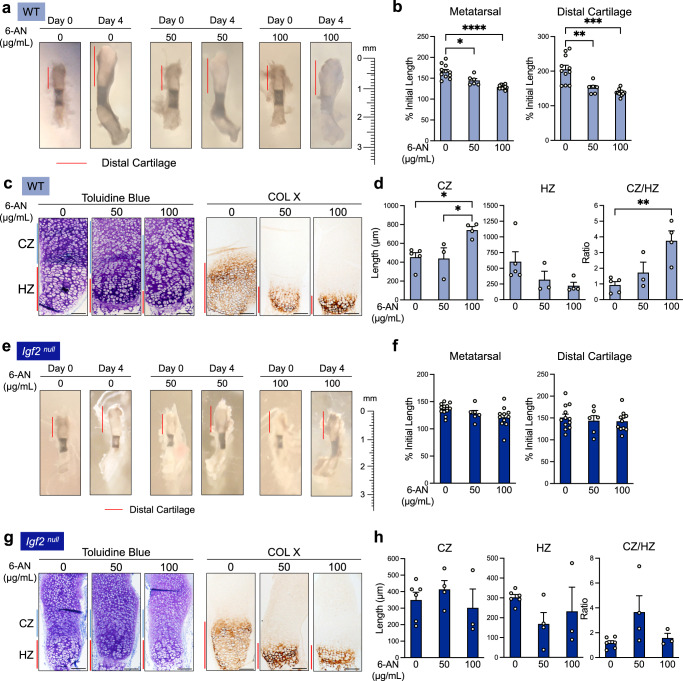
The pentose phosphate pathway is required for proper hypertrophy and bone growth in ex vivo metatarsal culture. **a** WT metatarsal explants cultured for 4 days in the presence or absence of 6-AN. Representative of *n* = 6–11 bones per group. **b** Quantification of metatarsal and distal cartilage growth from the beginning of the culture period (as percent of initial length of metatarsal or distal cartilage). *n* = 6–11 bones per group. **c** Toluidine blue staining for morphology and IHC for collagen X (hypertrophic marker) in ex vivo-cultured WT metatarsals treated with 6-AN. Representative of *n* = 3–5 bones per group. CZ columnar zone. HZ hypertrophic zone. **d** Measurements of the lengths of the CZ and HZ, and the ratio of the CZ to HZ, in ex vivo-cultured metatarsals treated with 6-AN. **e** *Igf2* null metatarsal explants cultured for 4 days with and without 6-AN. Representative of *n* = 6–12 bones per group. **f** Quantification of metatarsal and distal cartilage growth from the beginning of the culture period (as percent of initial length of metatarsal or distal cartilage). *n* = 6–12 bones per group. **g** Toluidine blue staining for morphology and IHC for collagen X in *Igf2* null metatarsals treated with 6-AN. Representative of *n* = 3–6 bones per group. **h** Measurements of the lengths of the CZ and HZ, and the ratio of the CZ to HZ, in *Igf2* null metatarsals treated with 6-AN. *n* = 3–6 bones per group. Scale bar: 100 µm. Data are presented as individual measurements with mean + SEM. Note that the control data (0 µg/mL 6-AN) for **b**, **d**, **f**, and **h** are also shown in Fig. 5. Statistical significance was calculated by Brown–Forsythe and Welch ANOVAs with Dunnett’s T3 test for multiple comparisons (**b**) or one-way ANOVA with Tukey’s test for multiple comparisons (**d**, **f**, **h**). *p*-values indicated as *p < 0.05, **p < 0.01, ***p < 0.001, ****p < 0.0001.

We next examined whether PPP activity was required for hypertrophic differentiation in intact bones using our ex vivo metatarsal culture system. Because a higher amount of 6-AN may be necessary to penetrate a metatarsal bone than would be necessary for monolayer-cultured chondrocytes, we included a 100 µg/mL concentration in the ex vivo culture. TUNEL assays confirmed that 6-AN treatment at this dose did not cause overt toxicity (Supplementary Fig. 3b). The overall longitudinal growth of the bone and distal cartilage were significantly reduced by 6-AN in a dose-dependent manner (Fig. 7a, b). Toluidine blue staining and Col X IHC indicated a significant lengthening of the CZ and a marked reduction in the length of the HZ (Fig. 7c, d). This resulted in a significantly increased ratio of CZ/HZ, thus accounting for the stunted bone growth (Fig. 7d). To confirm the specificity of this approach, we also used NADPH, which, as a product of the first steps in the PPP, is known to block G6PD activity^49,50^. NADPH treatment reduced ALP activity in chondrocytes, as well as cartilage growth and HZ length in metatarsals, mirroring the results from 6-AN (Supplementary Fig. 5). Thus, blocking the progression of the PPP significantly halted hypertrophic differentiation in chondrocytes, suggesting that the PPP is required for proper hypertrophic differentiation during cartilage development.

When 6-AN was applied to *Igf2* null metatarsal bones, the overall growth of the bone and distal cartilage was slightly reduced (Fig. 7e, f). Toluidine blue staining and Col X IHC indicated a trend of reduction in HZ length, but the changes in the lengths of the CZ and HZ were not as dramatic and were more variable than those seen in the WT bones (Fig. 7h, as compared to Fig. 7d). As inhibition of the PPP in *Igf2* null bones did not rescue the length of the CZ or HZ to match those of the WT bones, it suggests that IGF2 does not play a key role in regulating PPP activity in cartilage growth. Nevertheless, the PPP is still required for hypertrophy in the absence of IGF2.

## Discussion

**Fig. 8:**
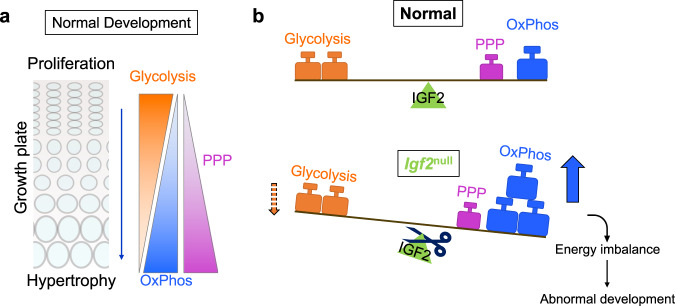
A working model of the metabolic switch in chondrocytes during hypertrophy. **a** As chondrocytes progress from a proliferating state to a hypertrophic state in the growth plate, they undergo a metabolic switch from a state of high glycolysis to a state of high oxidative phosphorylation and pentose phosphate pathway activity. **b** IGF2 balances the metabolic switch, such that when it is removed, hypertrophic *Igf2* null chondrocytes experience heightened oxidative phosphorylation compared to WT chondrocytes.

Chondrocytes consume glucose not only for energy, but also to use it as a building block for glycosaminoglycans (GAGs) in the extracellular matrix (ECM). They thus require a robust bioenergetic system. In this study, we demonstrated that a bioenergetic shift occurs during cartilage development in long bone growth. Our results suggest a working model in which glucose utilization shifts from glycolysis to OxPhos as chondrocytes mature from the proliferating to the hypertrophic stage, and this shift is accompanied by enhancement of the PPP (Fig. 8a). We discovered that distinct metabolic signatures are important for cartilage development, as both OxPhos and the PPP are required for chondrocyte maturation. Furthermore, IGF2 plays a pivotal role in balancing glucose metabolism by preventing overactive OxPhos, and perhaps exerts a lesser degree of control over glycolysis (Fig. 8b). The lack of IGF2 thus causes an imbalance between OxPhos and glycolysis.

There is a growing body of literature highlighting bioenergetic switching as a fundamental mechanism of developmental progression for a variety of cell types. For example, there is a shift from glycolysis to OxPhos upon neural stem cell differentiation^23^. This could be related to the hypoxic state in the stem cell niche and oxygen availability when differentiation ensues^23^. In contrast, during osteoblast differentiation, there is a switch from OxPhos to glycolysis, even though there is an abundance of oxygen^24,25,51^. Therefore, tissues have diverse strategies for energy balance during differentiation. During early stages of cartilage development, the tissue becomes increasingly hypoxic as the amount of ECM increases^52,53^, but chondrocytes subsequently have better access to blood vessels as hypertrophy progresses^54,55^. The switch from glycolysis to OxPhos during chondrocyte maturation may be supported by the change in this environmental niche.

A recent study showed that impairment of OxPhos, due to mitochondrial transcription factor A (*Tfam*) deficiency, resulted in a delay in hypertrophic maturation and shorter limbs, thus corroborating our result that OxPhos is required for hypertrophy^56^. Furthermore, HIF signaling balances OxPhos to support normal development^56,57^. Prior studies have used NADH fluorescence to gauge the level of glycolysis and OxPhos^20,58^. However, due to the limitations of fluorescence technology, it could not differentiate between the NADH generated by the mitochondrial TCA cycle and the NADH generated by cytoplasmic glycolysis. Our current study now provides a direct measurement of glycolysis and OxPhos in growth plate chondrocytes. Why is hypertrophy associated with increased OxPhos? Perhaps a dramatic escalation in protein and lipid synthesis is necessary to accommodate cell enlargement during hypertrophy^59^. Consistent with that, a higher level of mitochondrial protein was detected in hypertrophic chondrocytes, which may underpin the increase in baseline OCR and spare respiratory capacity in these cells^60^.

The PPP is a major pathway for controlling the cellular redox ratio, providing sugar intermediates for GAG synthesis, providing coenzymes (such as acetyl-CoA and NAD) for cellular processes, and nucleic acid synthesis^61–65^. We found that the activity of G6PD, a key enzyme in the PPP, was upregulated in hypertrophic chondrocytes. It has been shown that an increase in ROS leads to induction of G6PD, causing a rerouting of glucose to the PPP^42,66^. We therefore suspect that the increase in the PPP in hypertrophic chondrocytes is the result of coupling between the PPP and OxPhos to provide the necessary building blocks for GAG production, coenzymes for OxPhos, and as a countermeasure to prevent overt oxidative stress. In this study, we used the G6PD inhibitors 6-AN and NADPH to show that the PPP is indeed required for hypertrophy during normal cartilage development. However, the way in which the PPP achieves this role is not entirely clear. Since hypertrophy does not involve cell proliferation, it may serve to provide various pentose molecules for increased protein and lipid synthesis for cell enlargement^59^. It was previously shown that ROS promotes hypertrophy, which is consistent with the increased OxPhos in this zone^67^. As NADPH may counterbalance the effect of ROS, our result that excess NADPH suppressed hypertrophy lends further support to the notion that hypertrophy is enhanced by ROS.

In addition to uncovering the role of distinct metabolic signatures during normal chondrocyte maturation, our study also revealed a critical role for IGF2 signaling in balancing glycolysis and OxPhos (Fig. 8b). In our previous analysis, we had observed increases in glucose uptake, lactate production, and OCR in *Igf2* null chondrocytes. However, in that study, cells were not enriched to proliferating and hypertrophic populations. It is therefore not clear whether the result was skewed by the different proportions of proliferating and hypertrophic chondrocytes that might be present in WT and *Igf2* null populations. Furthermore, even if the proportion of cells at different stages were known, the levels of glycolysis and OxPhos in proliferating and hypertrophic chondrocytes under normal conditions were not clear.

In this study, we directly addressed this issue by forcing WT and *Igf2* null chondrocytes to have the same level of proliferation or hypertrophy via treatment with PTHrP or AA + BMP2. Thus, in our current study, there were no significant differences in markers for proliferation and hypertrophy between WT and *Igf2* null enriched chondrocytes, even though we observed aberrant chondrocyte hypertrophy in our previous study^20^. Our previous analysis using lactate and OCR assay kits showed that *Igf2* null chondrocytes had increased glucose uptake, glycolysis, and OxPhos, and that adding IGF2 back to cells remedied this effect. However, in that study, exogenous IGF2 did not alter glucose metabolism measurements in normal chondrocytes, suggesting that IGF2 is necessary for maintaining metabolic balance, but is not sufficient for altering overall glucose metabolism. In this study, we advanced our investigation by using the Seahorse metabolic analyzer to investigate glucose metabolism at both baseline and full capacity. We found that *Igf2* null hypertrophic chondrocytes had a higher level of OxPhos, which is consistent with our previous study and suggests that IGF2 is required to suppress OxPhos^20^. However, in this study, we did not observe any differences in glycolysis between WT and *Igf2* null proliferating or hypertrophic chondrocytes, contrary to what we found previously using the lactate assay kit^20^. It is possible that the previously observed increase in glycolysis in the *Igf2* null chondrocytes could be due to increased glucose uptake. Furthermore, in our previous study, cells were cultured for 4 days and were not glucose starved, while in the current Seahorse Glycolysis Stress Test, cells were starved of glucose for an hour and assessed after 20 min of glucose replenishment. It is therefore also possible that after 4 days of culture in our prior study, *Igf2* null chondrocytes had altered glycolysis even if glycolysis is not dramatically changed immediately after glucose treatment. Future experiments will include the assessment of glucose uptake and a time course of glycolysis after glucose depletion.

Although the increase in OxPhos seemed to be coupled to the increase in the PPP in WT hypertrophic chondrocytes, the additional increase in OxPhos in *Igf2* null hypertrophic chondrocytes was not matched by a further increase in the PPP. The increased OxPhos in *Igf2* null hypertrophic chondrocytes is consistent with the increase in ROS in *Igf2* null chondrocytes that we saw in our prior study^20^. Thus, there appears to be a defect in the coupling of OxPhos to the PPP in the absence of IGF2. This metabolic imbalance could underpin the phenotype of premature hypertrophy and growth restriction we have previously observed in *Igf2* null bones as postnatal development proceeds^20^. Strong IGF2 expression has been established in the proliferating and prehypertrophic zones^68^. It likely acts as a gatekeeper for the progression from the proliferating phase to the hypertrophic phase by controlling the balance of glucose metabolism. The absence of IGF2 could cause a partial relaxation of this gate, leading to elevated OxPhos that promotes hypertrophy and compromises the growth of the cartilage template. Finally, the fact that *Igf2* deficiency did not significantly alter OxPhos or glycolysis in proliferating chondrocytes could be related to the presence and spatial distribution of its potential receptors and IGF binding proteins (IGFBPs) in the developing cartilage^69–73^.

Apart from our study, other reports have shown the importance of IGF2 in glucose metabolism. IGF2 enhances OxPhos and reduces inflammation in macrophages; it also functions in osteoblast differentiation^74,75^. In contrast, IGF1 promotes glycolysis in neurons, multiple myeloma cells, and osteoblasts^24,76,77^. Thus, IGFs seem to regulate glucose metabolism in a context-dependent manner. One way that IGF2 controls glucose metabolism could be through the modulation of mitochondrial biogenesis, as has recently been shown in muscle, but this remains to be determined in chondrocytes^78^.

There are a few limitations of this study. First, the *Igf2* null mouse utilized here was a global knockout because a conditional mutant was unavailable at the time of the experiments. Although we chose the well-established ex vivo metatarsal culture model to assess the local effect of IGF2, future studies will utilize the conditional *Igf2* knockout mouse. Another limitation is the use of chemical inhibitors. While we confirmed our study with two well-established specific inhibitors for OxPhos and the PPP, and observed dose-dependent effects, we plan to perform genetic manipulation of glucose metabolism in the future to further solidify our findings. On the other hand, even if genetic manipulation is used, inhibition of one pathway may eventually affect another pathway because of feedback loops among the branches of glucose metabolism. A combination of radioactive carbon tracing and pathway perturbation should help to elucidate the dynamic balance of these pathways during cartilage development.

In summary, our investigation led to the discovery of distinct metabolic signatures and a bioenergetic switch in cartilage. Although glucose is widely used by most tissues, the preferential selection of its downstream metabolic pathways is critical for cell identity and function. Our work thus yields a fundamental yet largely unexplored mechanism of cartilage development and provides important insights into the pathogenesis of human abnormalities in skeletal growth such as Silver-Russell Syndrome.

## Materials and methods

### Experimental animals

All animal care and experimental procedures used in this study were approved by the Institutional Animal Care and Use Committee at Tufts University. Mice were housed under standard conditions (14-h light/10-h dark cycle, standard chow diet *ad libitum*).

### In vitro murine epiphyseal chondrocyte culture

Murine epiphyseal chondrocytes were isolated from the femur and tibia of postnatal day 6 (P6) mice based on a previously published protocol^79^. Chondrocytes were used at passage 2 and cultured in DMEM/F12 (Gibco #11330-032), 10% fetal bovine serum (FBS, Gibco #16000-004), and 1% antibiotic-antimycotic (Gibco #15240-062). Cells were seeded at a density of 1.5 × 10^4^ cells/cm^2^ in standard 24-well tissue culture plates for RT-PCR, EdU, and alkaline phosphatase assays. Treatment of the cells include: 10 ng/mL PTHrP (Peprotech #100-09), 50 µg/mL ascorbic acid (AA, Sigma #A4544) with 60 ng/mL BMP2 (R&D Systems #355-BM), 0.1 mM or 1.0 mM 3-nitropropionic acid (3-NPA, Sigma #N5636, in 0.01% DMSO), 0.2 µM or 0.3 µM each of rotenone and antimycin A (R/A, Sigma #R8875 and Sigma #A8674, in 0.03% DMSO), 10 µg/mL or 50 µg/mL 6-aminonicotinamide (6-AN, Alfa Aesar #L0669203, in 0.1% DMSO), 50 µM or 500 µM NADPH (Sigma #481973, in distilled-deionized water), or 10 ng/mL insulin-like growth factor 2 (IGF2, Peprotech #100-12). The corresponding amount of DMSO served as a control in each experiment.

### Ex vivo metatarsal bone culture

The middle three metatarsal bones were isolated from the hind paws of P0 mice and cultured for 4 days in DMEM (Gibco #11965-092) supplemented with 0.25% FBS, 50 µg/mL ascorbic acid, 1.25 mM sodium pyruvate (Sigma #P5280), and 1% antibiotic-antimycotic in the presence or absence of 1.0 mM or 3.0 mM 3-NPA (in 0.03% DMSO), 0.3 µM or 0.4 µM each of R/A (in 0.04% DMSO), 50 µg/mL or 100 µg/mL 6-AN (in 0.2% DMSO), or 500 µM or 2 mM NADPH (in distilled-deionized water). Bones were cultured on a rotating platform at 60 RPM to facilitate nutrient transfer. The corresponding amount of DMSO served as a control in each experiment.

### RT-qPCR

Total RNA was isolated with the RNeasy® Mini Kit (Qiagen #74106) or the PureLink™ RNA Mini Kit (Invitrogen #12183025) and reverse transcribed using M-MLV reverse transcriptase (Invitrogen #28025013). qPCR was performed using either the iQ5 Real Time Detection System (Bio-Rad) or the QuantStudio 6 Flex (Applied Biosystems). TATA-box binding protein (*Tbp*) served as the reference gene. Primer sequences can be found in Supplementary Table 1.

### EdU assay

EdU assays were performed using the Click-iT® Plus EdU Alexa Fluor® 488 Imaging Kit (Invitrogen #C10637). Epiphyseal chondrocytes enriched to proliferating (10 ng/mL PTHrP) or hypertrophic (50 µg/mL AA + 60 ng/mL BMP2) stages were treated with 10 µM 5-ethynyl-2’-deoxyuridine (EdU) in culture for 12 h, after which cells were fixed with 4% paraformaldehyde (PFA) for 15 min at 4 °C. For ex vivo cultured metatarsal bones, 10 µM EdU was added for 4 h prior to PFA fixation and decalcification in 0.3 M EDTA (Boston BioProducts #BM-150) with 0.0875% glycerol (Boston BioProducts #P-780). The click-chemistry reaction was performed according to the manufacturer’s instructions.

### Alkaline phosphate (ALP) activity assay

Alkaline phosphatase activity was assayed immediately after the EdU assay. Briefly, cells were washed 3 times with NTMT (100 mM sodium chloride, 100 mM Tris-HCl [pH 9.5], 50 mM magnesium chloride, 0.1% Tween-20) and stained with a combination of 250 µg/mL NBT (Roche #11383213001) and 125 µg/mL BCIP (Roche #1383221) in NTMT. DAPI (50 µg/mL in PBS) was applied for 20 minutes before imaging.

### Bioenergetic analysis

Glucose metabolism assays were performed using the Seahorse XFe96 metabolic analyzer (Agilent). Growth plate chondrocytes were seeded at 2–3 × 10^4^ cells per well in 96-well Seahorse assay plates that were pre-coated with 50 µg/mL poly-D-lysine (Millipore #A-003-E) for 1 h at 37 °C. For the Mito Stress Test Kit (Agilent #103015-100), cells were cultured for 4 days in DMEM/F12 supplemented with 10% FBS and 1% antibiotic-antimycotic before switching to Seahorse phenol red-free XF Base Medium (Agilent #103335-100) supplemented with 3.151 g/L glucose (Fluka #49159), 2.5 mM L-glutamine (Gibco #25030081), and 0.5 mM sodium pyruvate (Sigma-Aldrich #P5280). After baseline OCR and ECAR measurements, the following chemicals were added (all given as final in-well concentrations): Oligomycin (1 µM, Sigma #O4876), FCCP (0.5 µM, Sigma #C2920), rotenone (0.5 µM, Sigma #R8875)/antimycin A (0.5 µM, Sigma #A8674). For the Glycolysis Stress Test Kit (Agilent #103020-100), cells were cultured for 4 days in DMEM/F12 supplemented with 10% FBS and 1% antibiotic-antimycotic before switching to Seahorse phenol red-free XF Base Medium supplemented only with 2.5 mM L-glutamine. ECAR measurements were made at baseline and following treatment with glucose (10 mM), oligomycin (1 µM), and 2-deoxyglucose (2DG, 50 mM, Sigma #D8375). Data were normalized to protein content using a sulforhodamine B assay. Briefly, cells were fixed in 10% trichloroacetic acid (Sigma #T9159) for 30 min on ice and stained with 0.4% sulforhodamine B in 1% acetic acid for 20 min. Excess dye was removed by rinsing with 1% acetic acid, and bound dye was resuspended in 10 mM Tris, pH 10.5. Absorbance was measured at 515 nm. Calculations for Mito Stress Test (OCR) metrics: (1) spare respiratory capacity = OCR_FCCP_ – OCR_Baseline_, (2) ATP production = OCR_Baseline_ – OCR_Oligomycin_, (3) non-mitochondrial respiration = OCR_Rotenone/Antimycin A_. Calculations for Glycolysis Stress Test (ECAR) metrics: (4) glycolysis = ECAR_Glucose_ – ECAR_Baseline_, (5) glycolytic capacity = ECAR_Oligomycin_ – ECAR_Baseline_, (6) non-glycolytic acidification = ECAR_2-deoxyglucose_.

### Live/Dead toxicity assay

Epiphyseal chondrocytes were seeded at a density of 1.5 × 10^4^ cells/cm^2^ in standard 48-well tissue culture plates. Chondrocytes were treated with 3-NPA (0.1 mM or 1 mM in 0.01% DMSO) or 6-AN (10 µg/mL or 50 µg/mL in 0.1% DMSO) for 4 days and stained with the LIVE/DEAD™ Viability/Cytotoxicity Kit (Invitrogen #L3224) according to the manufacturer’s directions. Cells were quantified using MATLAB.

### Histology and immunohistochemistry

After PFA fixation and EDTA decalcification, metatarsal bones were embedded in paraffin and sectioned at 5 µm thickness. Sections were subjected to toluidine blue staining, Col X IHC, or TUNEL staining. For toluidine blue staining, deparaffinized sections were stained with a solution of 0.04% in 0.1 M sodium acetate (pH 4) for 10 min at room temperature. For Col X IHC, antigen retrieval was performed with 0.3% hyaluronidase (Sigma #H3506). Then sections were blocked with the M.O.M® Elite® Peroxidase Kit (Vector Laboratories #PK-2200) according to the manufacturer’s instructions and stained overnight with undiluted primary Col X antibody (gift from Drs. Thomas Linsenmayer and James Kubilus, deposited in the Developmental Studies Hybridoma Bank as X-AC9). The DAB Peroxidase (HRP) Substrate Kit (Vector Laboratories #SK-4100) was used for chromogenic detection. Incubation of sections with only secondary antibody served as negative controls. For TUNEL assays, sections were digested with 20 µg/mL proteinase K (VWR #0706) for 30 min at 37 °C. After washing with PBST, autofluorescence was quenched with 0.25% ammonium chloride in Tris-buffered saline twice for 5 min at room temperature. The tissues were then preincubated with terminal deoxynucleotidyl transferase (TdT) reaction buffer (25 mM Tris, 200 mM sodium cacodylate, 0.25 mg/mL bovine serum albumin, 1 mM cobalt chloride) for 10 min at room temperature. The TUNEL reaction was accomplished by the application of a mixture of 4 µM fluorescein-12-dUTP (Thermo Scientific #R0101) and 80 U/mL of TdT (Thermo Scientific #EP0161) in TdT reaction buffer for 1 h at 37 °C. Tissues were counterstained with DAPI. TUNEL-positive cells were quantified as a percentage of positive cells using a custom MATLAB script.

### Glucose 6-phosphate dehydrogenase activity assay

G6PD activity levels were assessed using a commercially available Glucose-6-Phosphate Dehydrogenase Activity Assay Kit (Cell Signaling Technology #12581 S) according to the manufacturer’s directions. Briefly, cultured chondrocytes were washed with ice-cold PBS and lysed for 5 min on ice with the kit’s 1x Cell Lysis Buffer supplemented with Protease Inhibitor Cocktail Set III (1 µL per 200 µL lysis buffer, Calbiochem #535140). The lysate was scraped from the wells and centrifuged for 10 min at 18,000 × *g* and 4 °C to pellet cell debris. The resulting supernatant was used for the G6PD assay. Samples were diluted 1:100 and plated for the assay according to the manufacturer’s instructions. The samples were incubated for 15 min at 37 °C prior to reading relative fluorescence units (RFUs) with excitation at 540 nm and emission at 590 nm on a SpectraMax M5 plate reader (Molecular Devices).

### Microscopy

Chondrocytes were imaged on an Olympus IX71 inverted microscope with an Olympus DP80 digital camera and associated software or on a Keyence BZ-X710 All-in-One fluorescence microscope. FIJI or MATLAB was used to automatically count EdU and DAPI-positive cells. Metatarsal bones were viewed under a Leica MZ16F stereomicroscope and images were taken with an iPhone SE mounted on the right eyepiece. Histological sections were imaged with an Olympus IX71 inverted microscope with an Olympus DP80 digital camera and associated software.

### Statistics and reproducibility

Each experiment was performed in triplicate, or as otherwise indicated. Outlier analysis for Seahorse experiments was performed by boxplot analysis in R version 3.5.0 “Joy in Playing” (outliers were defined as further than 1.5 * interquartile range away from the mean). Data in this paper are reported as individual measurements, with the mean shown where necessary. Error bars on graphs represent standard error of the mean (SEM). Data were analyzed by two-way ANOVA followed by Tukey’s test for multiple comparisons, one-way ANOVA followed by Sidak’s or Tukey’s multiple comparisons test, Brown–Forsythe and Welch ANOVAs with Dunnett’s T3 test for multiple comparisons, or unpaired two-tailed *t*-test using GraphPad Prism (version 8), as specified. *p* ≤ 0.05 was considered significant.

### Reporting summary

Further information on research design is available in the Nature Portfolio Reporting Summary linked to this article.

## Supplementary information

Supplementary Information

Description of Additional Supplementary Files

Supplementary Data 1

Reporting Summary

## Data Availability

The data generated during this study are available from the corresponding author upon request. The source data for graphs in the main text are available in Supplementary Data 1.

## Code availability

The custom code used to quantify live/dead cells and TUNEL-positive cells was written in MATLAB and is available on Github (https://github.com/canhelp/Cell-image).
