# Supplementary material for: A critical bioenergetic switch is regulated by IGF2 during murine cartilage development
Source: Commun Biol. 2022 Nov 11;5:1230. doi: 10.1038/s42003-022-04156-4 (PMC9652369; doi:10.1038/s42003-022-04156-4)
Supplement: Supplementary file 1 — Supplementary Information [file 42003_2022_4156_MOESM1_ESM.pdf]

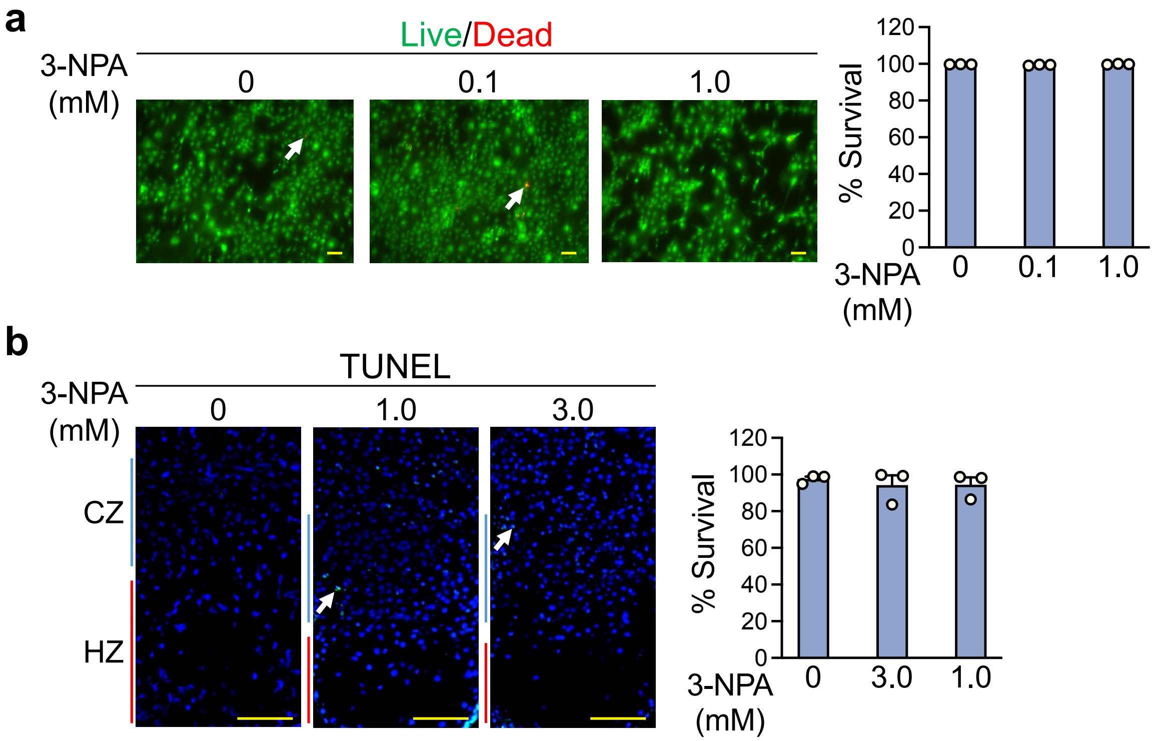

Supplementary Figure 1. 3-NPA does not cause overt toxicity in chondrocytes or *ex vivo*-cultured metatarsals. a Cytotoxicity of 3-NPA *in vitro* was assessed and quantified by Live/Dead assay in WT epiphyseal chondrocytes. Chondrocytes were cultured with 3-NPA for 4 days. Scale bar: 200 µm. **b** Cytotoxicity of 3-NPA in *ex vivo* WT metatarsal culture was assessed and quantified by TUNEL staining. Metatarsals were cultured with 3-NPA for 4 days. Scale bar: 100 µm. White arrows indicate dead cells. All data are mean + SEM, representative of n = 2 experiments with 3 wells or bones per group. None were significant by one-way ANOVA with Tukey's test for multiple comparisons.

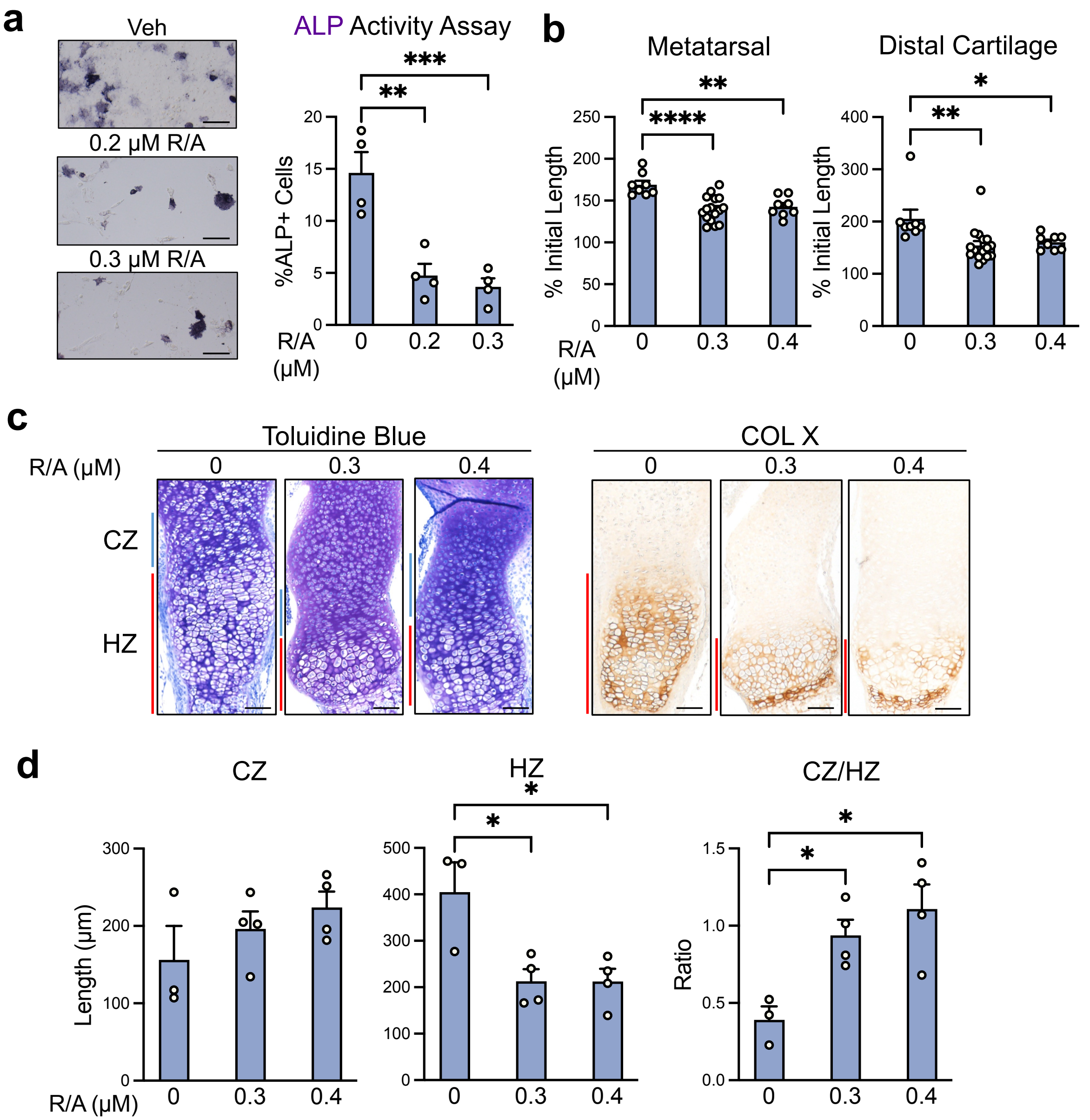

Supplementary Figure 2. Inhibition of oxidative phosphorylation by electron transport chain inhibitors rotenone and antimycin A (R/A) in normal chondrocytes inhibits hypertrophy and decreases the length of the hypertrophic zone. a Alkaline phosphatase (ALP) activity assay of WT hypertrophic chondrocytes treated with R/A. Representative of n = 3 experiments with 4 wells per group. Scale bar: 100 μm. **b** Quantification of metatarsal and distal cartilage growth from the beginning of the culture period (as percent of initial length of metatarsal or distal cartilage). n = 8-16 bones per group. **c** Toluidine blue staining and

hypertrophic marker collagen X IHC on *ex vivo*-cultured WT metatarsals treated with R/A. Representative of n = 3-4 bones per group. CZ, columnar zone. HZ, hypertrophic zone. **d** Measurements of the lengths of the CZ and HZ, and the ratio of the CZ to HZ, in *ex vivo*-cultured WT metatarsals treated with R/A. n = 3-4 bones per group. Scale bar: 100 µm. All data are mean + SEM. Statistical significance was calculated one-way ANOVA with Sidak's test for multiple comparisons (**a**), one-way ANOVA with Dunnett's test for multiple comparisons (**b**), and one-way ANOVA with Tukey's test for multiple comparisons (**d**). *p*-values indicated as * $p < 0.05$, ** $p < 0.01$, *** $p < 0.001$, **** $p < 0.0001$.

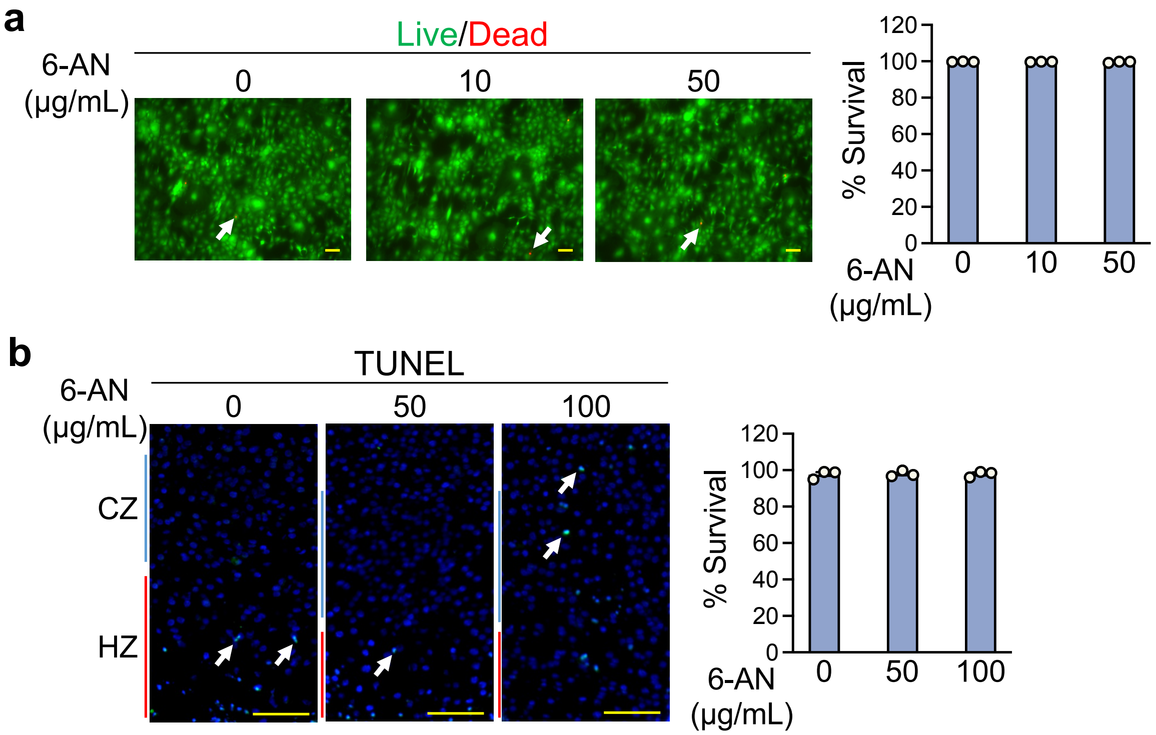

Supplementary Figure 3. 6-AN does not cause overt toxicity in chondrocytes or *ex vivo*-cultured metatarsals. a Cytotoxicity of 6-AN *in vitro* was assessed and quantified by Live/Dead assay in WT epiphyseal chondrocytes. Chondrocytes were cultured with 6-AN for 4 days. Scale bar: 200 µm. **b** Cytotoxicity of 6-AN in *ex vivo* WT metatarsal culture was assessed and quantified by TUNEL staining. Metatarsals were cultured with 6-AN for 4 days. Scale bar: 100 µm. White arrows indicate dead cells. All data are mean + SEM, representative of n = 2 experiments with 3 wells or bones per group. None were significant by one-way ANOVA with Tukey's test for multiple comparisons.

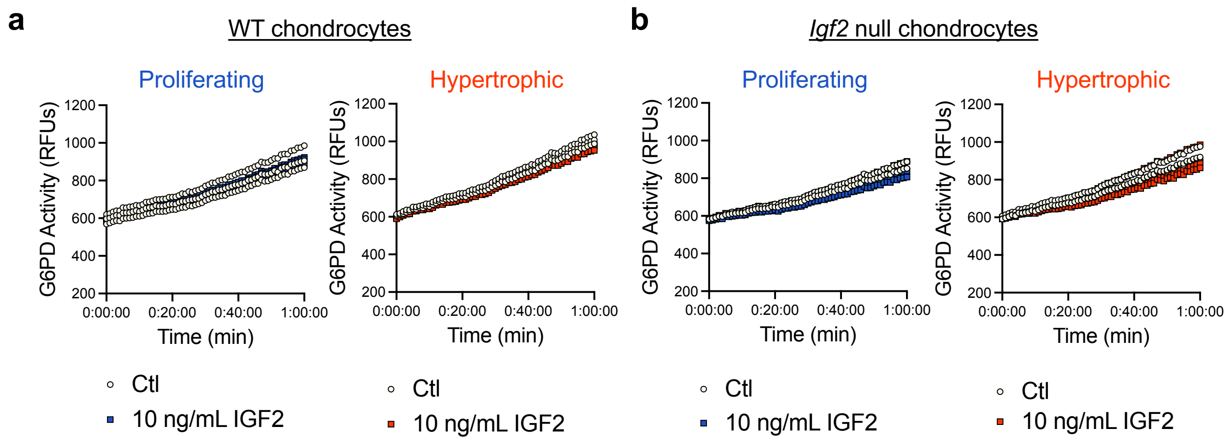

Supplementary Figure 4. G6PD activity is slightly reduced by exogenous IGF2. a G6PD activity in WT epiphyseal chondrocytes under proliferative and hypertrophic conditions, treated with or without 10 ng/mL IGF2. **b** G6PD activity in *Igf2* null epiphyseal chondrocytes under proliferative and hypertrophic conditions, treated with or without 10 ng/mL IGF2. n = 3 wells per group. Data are plotted as individual points at each time point. Statistical significance was calculated by unpaired two-tailed *t*-test at the last time point, and no statistical significance was found.

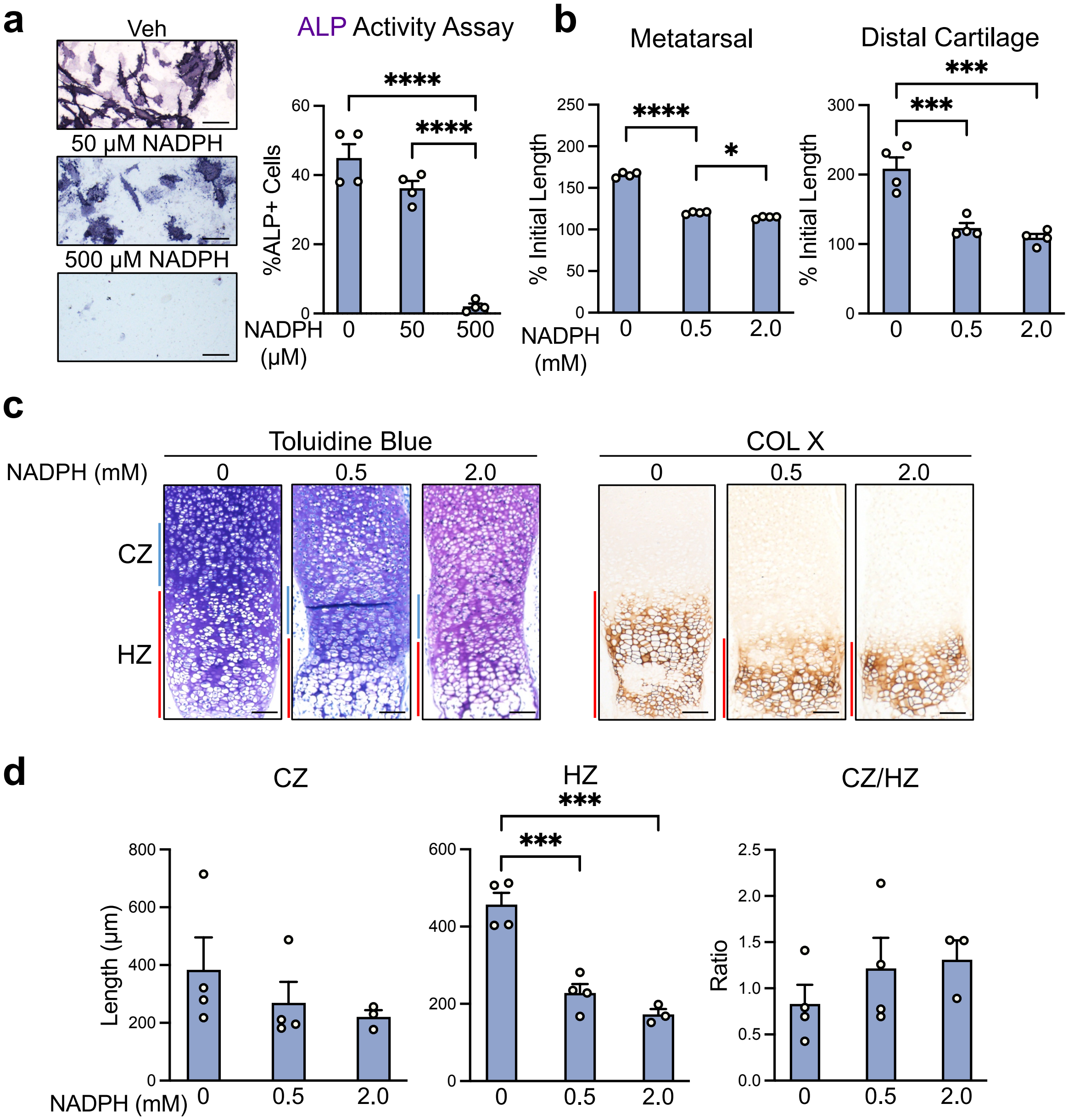

Supplementary Figure 5. Treatment of normal chondrocytes with NADPH inhibits hypertrophy and decreases the length of the hypertrophic zone. a Alkaline phosphatase (ALP) activity assay of WT hypertrophic chondrocytes treated with NADPH, which inhibits multiple steps in the pentose phosphate pathway. Representative of n = 3 experiments with 4 wells per group. Scale bar: 100 µm. **b** Quantification of metatarsal and distal cartilage growth from the beginning of the culture period (as percent of initial length of metatarsal or distal cartilage). n = 4 bones per group. **c** Toluidine blue staining and collagen X (hypertrophic

marker) IHC on *ex vivo*-cultured WT metatarsals treated with NADPH. Representative of n = 3-4 bones per group. CZ, columnar zone. HZ, hypertrophic zone. **d** Measurements of the lengths of the CZ and HZ, and the ratio of the CZ to the HZ, in *ex vivo*-cultured WT metatarsals treated with NADPH. Scale bar: 100 µm. n = 3-4 bones per group. All data are mean + SEM. Statistical significance was calculated one-way ANOVA with Tukey's test for multiple comparisons (**a**, **b**, **d**). *p*-values indicated as * $p < 0.05$, ** $p < 0.01$, *** $p < 0.001$, **** $p < 0.0001$.

Supplementary Table 1. Primer Information

Primer	Sequence	Citation (if available)
Col2a1 Fwd	5’ – ACA TAG GGC CTG TCT GCT TCT TGT – 3’	
Col2a1 Rev	5’ – TGA CTG CGG TTG GAA AGT GTT TGG – 3’	
Acan Fwd	5’ – GAG ACT TCT GCC TCT GGA ATA G – 3’	
Acan Rev	5’ – CTC CAG AAG GAA TCC CAC TAA C – 3’	
Alp Fwd	5’ – CCA ACT CTT TTG TGC CAG AGA – 3’	PrimerBank [1-3] ID 6671533a1
Alp Rev	5’ – GGC TAC ATT GGT GTT GAG CTT TT – 3’	PrimerBank [1-3] ID 6671533a1
Tbp Fwd	5’ – CTA CCG TGA ATC TTG GCT GTA A – 3’	
Tbp Rev	5’ – GTT GTC CGT GGC TCT CTT ATT – 3’	

Supplementary References

1. Wang, X. and Seed, B. A PCR primer bank for quantitative gene expression analysis. Nucleic Acids Research. **31**, e154; 10.1093/nar/gng154 (2003).

2. Spandidos, A., Wang, X., Wang, H., Dragnev, S., Thurber, T. and Seed, B. A comprehensive collection of experimentally validated primers for Polymerase Chain Reaction quantitation of murine transcript abundance. BMC Genomics. **9**, 633; 10.1186/1471-2164-9-633 (2008).

3. Spandidos, A., Wang, X., Wang, H. and Seed, B. PrimerBank: a resource of human and mouse PCR primer pairs for gene expression detection and quantification. Nucleic Acids Research. **38**, D792-D799 (2010).
