# Supplementary material for: A critical bioenergetic switch is regulated by IGF2 during murine cartilage development
Source: Commun Biol. 2022 Nov 11;5:1230. doi: 10.1038/s42003-022-04156-4 (PMC9652369; doi:10.1038/s42003-022-04156-4)
Supplement: Supplementary file 4 — Reporting Summary [file 42003_2022_4156_MOESM4_ESM.pdf]

nature portfolio

Corresponding author(s): Li Zeng

Last updated by author(s): Oct 16, 2022

Reporting Summary

Nature Portfolio wishes to improve the reproducibility of the work that we publish. This form provides structure for consistency and transparency in reporting. For further information on Nature Portfolio policies, see our Editorial Policies and the Editorial Policy Checklist.

Statistics

For all statistical analyses, confirm that the following items are present in the figure legend, table legend, main text, or Methods section.

n/a	Confirmed	
☐	☒	The exact sample size (*n*) for each experimental group/condition, given as a discrete number and unit of measurement
☐	☒	A statement on whether measurements were taken from distinct samples or whether the same sample was measured repeatedly
☐	☒	The statistical test(s) used AND whether they are one- or two-sided<br>*Only common tests should be described solely by name; describe more complex techniques in the Methods section.*
☐	☒	A description of all covariates tested
☐	☒	A description of any assumptions or corrections, such as tests of normality and adjustment for multiple comparisons
☐	☒	A full description of the statistical parameters including central tendency (e.g. means) or other basic estimates (e.g. regression coefficient) AND variation (e.g. standard deviation) or associated estimates of uncertainty (e.g. confidence intervals)
☐	☒	For null hypothesis testing, the test statistic (e.g. *F*, *t*, *r*) with confidence intervals, effect sizes, degrees of freedom and *P* value noted<br>*Give P values as exact values whenever suitable.*
☒	☐	For Bayesian analysis, information on the choice of priors and Markov chain Monte Carlo settings
☒	☐	For hierarchical and complex designs, identification of the appropriate level for tests and full reporting of outcomes
☒	☐	Estimates of effect sizes (e.g. Cohen's *d*, Pearson's *r*), indicating how they were calculated

Our web collection on statistics for biologists contains articles on many of the points above.

Software and code

Policy information about availability of computer code

Data collection	G6PD assay data was collected using a SpectraMax M5 plate reader and SoftMax Pro version 4.8. Chondrocytes were imaged using an Olympus IX71 inverted microscope with an Olympus DP80 camera and cellSens version 1.18, or a Keyence BZ-X710 All-in-One fluorescence microscope and BZ-X Viewer software. EdU positive cells were quantified using FIJI version 1.53q. Live/dead cells and TUNEL-positive cells were quantified using MATLAB and is available on Github (https://github.com/canhelp/Cell-image).
Data analysis	Outliers analysis for Seahorse experiments were identified in R version 3.5.0 "Joy in Playing" and defined as points further than 1.5 * interquartile range away from the mean (script available from the corresponding author upon request. Statistical analysis was performed in GraphPad Prism version 8.

For manuscripts utilizing custom algorithms or software that are central to the research but not yet described in published literature, software must be made available to editors and reviewers. We strongly encourage code deposition in a community repository (e.g. GitHub). See the Nature Portfolio guidelines for submitting code & software for further information.

Data

Policy information about availability of data

All manuscripts must include a data availability statement. This statement should provide the following information, where applicable:

- Accession codes, unique identifiers, or web links for publicly available datasets
- A description of any restrictions on data availability
- For clinical datasets or third party data, please ensure that the statement adheres to our policy

The data used to generate graphs can be found in the Supplementary Data 1 file. All other data generated during this study are available from the corresponding author upon request.

Human research participants

Policy information about studies involving human research participants and Sex and Gender in Research.

Reporting on sex and gender	This study does not involve human research subjects.
Population characteristics	This study does not involve human research subjects.
Recruitment	This study does not involve human research subjects.
Ethics oversight	This study does not involve human research subjects.

Note that full information on the approval of the study protocol must also be provided in the manuscript.

Field-specific reporting

Please select the one below that is the best fit for your research. If you are not sure, read the appropriate sections before making your selection.

☒ Life sciences ☐ Behavioural & social sciences ☐ Ecological, evolutionary & environmental sciences

For a reference copy of the document with all sections, see nature.com/documents/nr-reporting-summary-flat.pdf

Life sciences study design

All studies must disclose on these points even when the disclosure is negative.

Sample size	Sample sizes were chosen based on prior experience from our laboratory.
Data exclusions	Outliers were excluded in Seahorse experiments. Outliers were defined as further than 1.5 * the interquartile range away from the mean. Additional wells were excluded that did not respond to drug injections.
Replication	Figures present representative data. The number of experimental replicates and/or technical replicates (wells or bones) is indicated in the figure legends.
Randomization	No randomization was used in this study.
Blinding	Investigators were not blinded to group allocation during collection, but were blinded to group allocation during analysis.

Reporting for specific materials, systems and methods

We require information from authors about some types of materials, experimental systems and methods used in many studies. Here, indicate whether each material, system or method listed is relevant to your study. If you are not sure if a list item applies to your research, read the appropriate section before selecting a response.

Materials & experimental systems

n/a	Involved in the study	
☐	☒	Antibodies
☐	☒	Eukaryotic cell lines
☒	☐	Palaeontology and archaeology
☐	☒	Animals and other organisms
☒	☐	Clinical data
☒	☐	Dual use research of concern

Methods

n/a	Involved in the study	
☒	☐	ChIP-seq
☒	☐	Flow cytometry
☒	☐	MRI-based neuroimaging

Antibodies

Antibodies used	Anti-collagen 10 antibody was a kind gift from Drs. Thomas Linsenmayer and James Kubilus (Tufts University). This antibody was deposited in the Developmental Studies Hybridoma Bank (DSHB Hybridoma Product X-AC9) by Dr. Linsenmayer.
Validation	This antibody has been validated in chicken, cuttlefish, human, mouse, and zebrafish. Further information and citations are available on the DSHB website (https://dshb.biology.uiowa.edu/X-AC9).

Eukaryotic cell lines

Policy information about cell lines and Sex and Gender in Research

Cell line source(s)	Primary chondrocytes were isolated from male and female WT and Igf2 null littermates at postnatal day 6.
Authentication	Cells were not authenticated.
Mycoplasma contamination	Cells were not tested for mycoplasma contamination.
Commonly misidentified lines (See ICLAC register)	No commonly misidentified cell lines were used in this study.

Animals and other research organisms

Policy information about studies involving animals; ARRIVE guidelines recommended for reporting animal research, and Sex and Gender in Research

Laboratory animals	The mice used in this study are Igf2 null and WT on a C57BL/6 background. Mice were used at postnatal day 0 (metatarsal culture) and postnatal day 6 (chondrocyte isolation). Both male and female mice were used.
Wild animals	This study did not involve wild animals.
Reporting on sex	Cells and bones from both male and female mice were used. We did not investigate differences due to sex because we focused on growth in newborn animals within 7 days of birth. During this period, we do not expect sexual dimorphism in growth.
Field-collected samples	This study did not involve field-collected samples.
Ethics oversight	All animal care and experimental procedures used in this study were approved by the Institutional Animal Care and Use Committee at Tufts University.

Note that full information on the approval of the study protocol must also be provided in the manuscript.
